# Supplementary material for: Effects of Household Processing on Residues of the Chiral Fungicide Mandipropamid in Four Common Vegetables
Source: Int J Environ Res Public Health. 2022 Nov 23;19(23):15543. doi: 10.3390/ijerph192315543 (PMC9735481; doi:10.3390/ijerph192315543)
Supplement: Supplementary file 1 [file ijerph-19-15543-s001.zip › ijerph-2017260-supplementary.pdf]

# Supporting information

**Table S1.** Recoveries and relative standard deviations (RSDs) of mandipropamid enantiomers in different parts of tested vegetable samples.

| Matrix | Analyte | Part | Spiked level (μg/kg) | Average recovery, RSD (%, *n* = 5) |
|---|---|---|---|---|
| Tomato | *R* | Whole | 2.5 | 96.1, 2.7 |
| | | | 25 | 96.1, 0.2 |
| | | | 500 | 97.0, 4.4 |
| | | Skin | 2.5 | 91.0, 9.7 |
| | | | 25 | 89.2, 8.2 |
| | | | 500 | 87.1, 5.1 |
| | | Pulp | 2.5 | 89.5, 1.8 |
| | | | 25 | 89.9, 9.9 |
| | | | 500 | 90.4, 6.1 |
| | | Puree | 2.5 | 91.4, 7.8 |
| | | | 25 | 90.9, 3.7 |
| | | | 500 | 89.9, 5.9 |
| | | Juice | 2.5 | 84.9, 1.8 |
| | | | 25 | 82.6, 4.3 |
| | | | 500 | 86.6, 2.9 |
| | *S* | Whole | 2.5 | 97.2, 9.8 |
| | | | 25 | 96.5, 3.6 |
| | | | 500 | 97.7, 2.8 |
| | | Skin | 2.5 | 96.0, 4.0 |
| | | | 25 | 95.9, 2.3 |
| | | | 500 | 95.5, 3.4 |
| | | Pulp | 2.5 | 89.9, 10.1 |
| | | | 25 | 90.0, 9.7 |
| | | | 500 | 89.7, 10.1 |
| | | Puree | 2.5 | 97.0, 11.5 |
| | | | 25 | 99.6, 9.2 |
| | | | 500 | 97.5, 3.5 |
| | | Juice | 2.5 | 89.8, 6.4 |
| | | | 25 | 90.7, 10.2 |
| | | | 500 | 92.1, 7.0 |
| Cucumber | *R* | Whole | 2.5 | 93.2, 8.0 |
| | | | 25 | 94.5, 8.9 |
| | | | 500 | 92.4, 6.3 |
| | | Skin | 2.5 | 92.0, 10.8 |
| | | | 25 | 92.9, 10.0 |
| | | | 500 | 98.7, 5.6 |
| | | Pulp | 2.5 | 95.8, 2.3 |
| | | | 25 | 97.0, 2.7 |
| | | | 500 | 92.5, 8.8 |
| | | Puree | 2.5 | 100.4, 4.8 |
| | | | 25 | 96.5, 3.0 |
| | | | 500 | 96.9, 3.6 |
| | | Juice | 2.5 | 97.0, 1.3 |
| | | | 25 | 97.6, 4.3 |
| | | | 500 | 97.4, 5.4 |

| | | | | |
|---|---|---|---|---|
| | *S* | Whole | 2.5 | 88.7, 13.1 |
| | | | 25 | 92.8, 9.1 |
| | | | 500 | 91.9, 10.7 |
| | | Skin | 2.5 | 90.8, 6.7 |
| | | | 25 | 94.3, 3.9 |
| | | | 500 | 94.3, 3.1 |
| | | Pulp | 2.5 | 90.6, 7.0 |
| | | | 25 | 97.9, 7.5 |
| | | | 500 | 94.7, 2.4 |
| | | Puree | 2.5 | 91.5, 10.1 |
| | | | 25 | 91.6, 7.7 |
| | | | 500 | 100.8, 4.0 |
| | | Juice | 2.5 | 97.5, 1.3 |
| | | | 25 | 90.8, 9.3 |
| | | | 500 | 92.2, 8.4 |
| Chinese cabbage | *R* | Whole | 2.5 | 101.0, 4.6 |
| | | | 25 | 99.4, 4.1 |
| | | | 500 | 96.4, 6.8 |
| | *S* | Whole | 2.5 | 96.6, 7.9 |
| | | | 25 | 96.0, 6.1 |
| | | | 500 | 97.1, 4.2 |
| Cowpea | *R* | Whole | 2.5 | 97.3, 2.9 |
| | | | 25 | 97.0, 4.1 |
| | | | 500 | 93.1, 7.2 |
| | *S* | Whole | 2.5 | 89.8, 4.8 |
| | | | 25 | 98.8, 4.9 |
| | | | 500 | 98.4, 3.6 |

**Table S2.** Residues of mandipropamid enantiomers and racemate in brine at different intervals after pickling cucumber, Chinese cabbage and cowpea samples.

| **Matrix** | **Interval (d)** | **Residues (μg/L, average value ± SD, *n* = 3)** | | |
|---|---|---|---|---|
| | | ***R*** | ***S*** | ***Rac*** |
| Cucumber | 0 | 0.26 ± 0.01 | 0.23 ± 0.01 | 0.59 ± 0.03 |
| | 1 | 0.39 ± 0.01 | 0.42 ± 0.01 | 0.81 ± 0.02 |
| | 3 | 0.48 ± 0.01 | 0.50 ± 0.01 | 0.98 ± 0.03 |
| | 5 | 0.65 ± 0.01 | 0.67 ± 0.01 | 1.32 ± 0.03 |
| | 7 | 1.26 ± 0.03 | 1.34 ± 0.01 | 2.60 ± 0.05 |
| | 14 | 0.61 ± 0.04 | 0.69 ± 0.02 | 1.30 ± 0.07 |
| | 21 | 0.55 ± 0.02 | 0.65 ± 0.03 | 1.20 ± 0.06 |
| Chinese cabbage | 0 | 0.01 ± 0.00 | 0.02 ± 0.00 | 0.01 ± 0.00 |
| | 1 | 0.01 ± 0.00 | 0.01 ± 0.00 | 0.02 ± 0.00 |
| | 3 | 0.02 ± 0.00 | 0.01 ± 0.00 | 0.03 ± 0.00 |
| | 5 | 0.03 ± 0.00 | 0.01 ± 0.00 | 0.04 ± 0.01 |
| | 7 | 0.05 ± 0.01 | 0.02 ± 0.01 | 0.07 ± 0.02 |
| | 14 | 0.04 ± 0.00 | 0.02 ± 0.00 | 0.06 ± 0.01 |
| | 21 | 0.02 ± 0.01 | 0.01 ± 0.00 | 0.03 ± 0.02 |
| Cowpea | 0 | 0.01 ± 0.00 | 0.01 ± 0.00 | 0.02 ± 0.00 |
| | 1 | 0.01 ± 0.00 | 0.01 ± 0.00 | 0.02 ± 0.00 |
| | 3 | 0.01 ± 0.00 | 0.01 ± 0.00 | 0.02 ± 0.00 |
| | 5 | 0.04 ± 0.01 | 0.05 ± 0.00 | 0.09 ± 0.02 |
| | 7 | 0.22 ± 0.05 | 0.28 ± 0.07 | 0.50 ± 0.13 |
| | 14 | 0.01 ± 0.00 | 0.01 ± 0.00 | 0.02 ± 0.00 |
| | 21 | 0.01 ± 0.00 | 0.02 ± 0.01 | 0.03 ± 0.01 |
